# Clinical Serum-Anchored Computational Design Pipeline for a Broad-Spectrum Influenza Multi-Epitope mRNA Vaccine

**DOI:** 10.3390/biology15040357

**Published:** 2026-02-19

**Authors:** Lifang Yuan, Zhiyao Ouyang, Yifan Zhao, Rongjun Bi, Yanjing Wu, Xu Li, Yingrui Li, Jiaping Song, Wei Li, Mingchen Yan, Simin Wen, Huanle Luo, Tian Bai, Yuelong Shu, Yongkun Chen

**Affiliations:** 1 Guangdong Provincial Key Laboratory of Infection Immunity and Inflammation, School of Basic Medical Sciences, Shenzhen University Medical School, Shenzhen University, Shenzhen 518060, China; yuanlf@szu.edu.cn (L.Y.); 2500243015@mails.szu.edu.cn (Y.W.); 2 Guangdong Key Laboratory for Biomedical Measurements and Ultrasound Imaging, National-Regional Key Technology Engineering Laboratory for Medical Ultrasound, School of Biomedical Engineering, Shenzhen University Medical School, Shenzhen 518060, China; 3 School of Public Health (Shenzhen), Shenzhen Key Laboratory of Pathogenic Microbes and Biosafety, Shenzhen Campus of Sun Yat-sen University, Shenzhen 518107, China; ouyzhy6@mail2.sysu.edu.cn (Z.O.); zhaoyf65@mail2.sysu.edu.cn (Y.Z.); birj@mail2.sysu.edu.cn (R.B.); luohle@mail.sysu.edu.cn (H.L.); 4 Department of Pathogenic Biology and Immunology, School of Basic Medical Science, Xiangnan University, Chenzhou 423000, Hunan, China; lixu@xnu.edu.cn; 5 Shenzhen Digital Life Institute, Shenzhen 518000, Guangdong, China; liyr@icarbonx.com (Y.L.); songjiaping@icarbonx.com (J.S.); liwei@icarbonx.com (W.L.); mcyan90@gmail.com (M.Y.); 6 Guangzhou First People’s Hospital, Guangzhou Medical University, Guangzhou 510180, China; realmint@163.com; 7 Key Laboratory of Tropical Disease Control (Sun Yat-sen University), Ministry of Education, Guangzhou 510080, China; 8 Key Laboratory of Pathogen Infection Prevention and Control (MOE), State Key Laboratory of Respiratory Health and Multimorbidity, National Institute of Pathogen Biology, Chinese Academy of Medical Sciences and Peking Union Medical College, Beijing 102629, China; baitian@ipbcams.ac.cn

**Keywords:** influenza, broad-spectrum vaccine, peptide-based vaccine, multiepitope, antibody-peptide microarray, immunoinformatics

## Abstract

Influenza’s pandemic threat is driven by antigenic drift, which limits the efficacy of conventional vaccines. To address this challenge, we established a clinical serum-anchored computational design pipeline for a broad-spectrum multi-epitope mRNA vaccine (MEMV), bridging the gap between pure in silico design and clinical applicability. Using 36 longitudinal sera (d0/d28/d365) from 12 well-characterized human cohorts (6 vaccine recipients and 6 influenza patients) and high-density antibody-peptide microarrays, we empirically identified 12 immunodominant B-cell linear epitopes from the nucleoprotein (NP) of influenza A (H1N1/H3N2) and B viruses. These experimentally validated epitopes were combined with in silico-predicted conserved helper T-lymphocyte (HTL)/cytotoxic T-lymphocyte (CTL) epitopes (from NP/HA/NA) to construct MEMVs candidates, ensuring high antigenicity, non-toxicity, and 95.63% global HLA coverage. Molecular docking and 100 ns molecular dynamics (MD) simulations confirmed favorable conformational compatibility between MEMVs and Toll-like receptor 3 (TLR3) in silico immunization via C-ImmSim predicted robust B/T-cell responses and protective cytokine (IFN-γ/IL-10) production. Collectively, this pipeline shortens the preliminary design cycle for influenza vaccines, provides a standard epitope-combination strategy, and offers direct targets for follow-up in vitro/in vivo experiments.

## Simple Summary

Influenza remains a major global health threat because the virus mutates constantly, often making traditional vaccines less effective. To address this challenge, this study developed a new method for designing "broad-spectrum" mRNA vaccines that protect against multiple influenza virus strains. Unlike previous methods that relied entirely on computer predictions, this approach used real blood samples from vaccinated people and from patients with influenza to identify specific epitopes that trigger a strong and long-lasting immune response. By combining these real-world biological markers with advanced computer modeling, the researchers constructed three new vaccine candidates. Computer simulations confirmed that these vaccine candidates are safe, stable, and capable of covering approximately 95.63% of the global population. This “clinical serum-anchored” design pipeline bridges the gap between theoretical design and real-world application, offering a faster and more reliable way to develop effective vaccines for future flu outbreaks.

## 1. Introduction

Influenza viruses (types A–D) cause acute respiratory infections, with annual global tolls of 300,000–500,000 deaths and 3–5 million severe cases [1]. Seasonal outbreaks in humans are primarily driven by influenza A (IAV, notably H1N1/H3N2 subtypes) and B (IBV, B/Victoria/B/Yamagata lineages) viruses [2]. As RNA viruses with segmented genomes, they encode key proteins including hemagglutinin (HA), neuraminidase (NA), and nucleoprotein (NP) [3,4].

Vaccination remains the most effective strategy for influenza prevention, but it faces significant efficacy challenges [5]. Traditional vaccines rely on strain matching, yet viral antigenic changes often lead to mismatches, resulting in an effectiveness of only 10–60% [6]. This underscores the need for broad-spectrum vaccine design strategies that target diverse IAV/IBV subtypes.

Recently, peptide-based vaccines (which induce both humoral and T-cell responses [7]) and mRNA vaccines (offering speed, flexibility, and safety [8]) have emerged as promising platforms. Computational prediction of immunodominant epitopes has further enabled the rapid development of multi-epitope vaccines against various pathogens [9,10,11]. However, two critical gaps remain in current design workflows: (1) purely in silico studies lack empirical validation of epitope immunogenicity in humans, leading to potential discrepancies between predicted and in vivo efficacy, and (2) existing experimental and computational studies rarely use longitudinal clinical sera (pre-/post-vaccination/infection) to capture durable epitope responses, limiting the translation of candidate vaccines to long-term protection.

To address these gaps, this study aimed to establish a clinical serum-anchored computational design pipeline for broad-spectrum multiepitope mRNA vaccines (MEMVs) against seasonal influenza viruses. To this end, we first empirically identified immunodominant B-cell linear epitopes of NP from vaccinated/infected humans via antibody-peptide microarrays (a gold-standard tool for serological epitope mapping [12]). We used 36 longitudinal sera (d0/d28/d365) from 12 well-characterized cohorts (6 quadrivalent inactivated influenza vaccine recipients and 6 laboratory-confirmed influenza patients), ensuring the selected B-cell linear epitopes are not just computationally predicted but functionally reactive in human hosts and capable of inducing long-lived immunity. We then complemented these experimentally validated NP epitopes with in silico-predicted HTL/CTL epitopes (from NP) and previously validated HA/NA epitopes to construct MEMV candidates. This workflow merges the real-world relevance of clinical data with the efficiency of computational optimization, aiming to provide a reusable design framework for subsequent influenza vaccine development. Preliminary in silico assessments showed promising characteristics of the pipeline, with further experimental trials needed to validate the efficacy and cross-protection of MEMV candidates derived from this pipeline.

## 2. Materials and Methods

### 2.1. Ethics and Experiment Samples

Novel epitopes were identified using samples from our prior research [13]: 6 Quadrivalent Inactivated Influenza Vaccine (QIV)-vaccinated volunteers and 6 influenza patients enrolled at Sun Yat-sen University (China, November 2020–December 2021) with ethics approval (No. 2020045; approval date: 25 October 2020) and written informed consent. To eliminate the interference of pre-existing immunity and ensure the specificity of epitope-induced immune responses, all participants were screened based on strict inclusion criteria: no history of influenza infection or vaccination within the past three years before enrollment. For vaccinated individuals, 18 blood samples were collected pre-vaccination (d0), 28 days post-vaccination (d28), and 365 days post-vaccination (d365); for patients, 18 samples were collected at diagnosis (d0), 28 days post-diagnosis (d28), and 365 days post-diagnosis (d365). Serum was separated immediately and stored at −80 °C.

### 2.2. Selection of Antigens and Utilized Databases

NP sequences of 2020–2021 QIV strains (H1N1 A/Guangdong-Maonan/SWL1536/2019, H3N2 A/Hong Kong/2671/2019, B/Vic B/Washington/02/2019, B/Yam B/Phuket/3073/2013) were retrieved from GISAID [14] (accessions EPI1716625, EPI1698482, EPI1422445, EPI544260). Full-length NP sequences of prevalent human H1N1, H3N2, and IBV strains were downloaded from the Influenza Virus Database [15]. Epitope validation was performed using the Immune Epitope Database (IEDB, supported by NIAID); the Toll-like receptor 3 (TLR3) PDB file (PDB ID: 2A0Z) was obtained from NCBI MMDB.

### 2.3. Epitope Identification by Antibody-Peptide Microarray

This section describes the core experimental workflow for epitope identification—antibody–peptide microarrays were used to screen differential B-cell linear epitopes from clinical sera, providing the empirical basis for subsequent bioinformatic selection of vaccine candidates. Epitope identification/evaluation followed our prior work [13] and Aaron Arvey’s protocol [12]. The microarray included 10-amino acid peptides from infectious/autoimmune antigens and 4000 human extracellular/secreted proteins. We aimed to identify NP “potent peptides” (d28 vs. d0) and “long-lived peptides” (d365 vs. d0) linked to QIV vaccination or infection, which helped capture epitopes with both short-term immunogenicity and long-term persistence.

### 2.4. Selection of B-Cell Linear Epitopes

B-cell linear epitopes of NP were retrieved from the IEDB database, and 10-amino acid epitopes identified by peptide microarray detection (the experimentally validated candidates) were mapped to these IEDB-annotated epitopes. Correctly mapped epitopes were aligned with full-length NP protein sequences, and both well-aligned and unmapped epitopes (all experimentally derived) were selected for further bioinformatic analysis.

### 2.5. Prediction of HTL Epitopes

The IEDB major MHC II server [16] (NetMHCIIPan 4.1 EL; 15-mer; 7-allele reference set [17]) was used to predict binders with a percentile rank < 0.5. Candidates were filtered based on: antigenicity > 0.5 (VaxiJen v2.0 [18]), non-allergenic (AllerTOP v.2.0 [19]), non-toxic (ToxinPred) [20], and whether they were IFN-γ-inducible (IFN-γ Epitope Server) [21].

### 2.6. Prediction of CTL Epitopes

The IEDB MHC I server [22] (NetMHCpan 4.1 EL; 9-/10-mer; 12 prevalent HLA-I alleles [23]) was used to select binders with percentile rank <0.5. Final epitopes met the following criteria: antigenicity > 1.0 (VaxiJen v2.0 [18]), non-allergenic (AllerTOP v.2.0 [19]), non-toxic (ToxinPred) [20], immunogenicity score > 0 (IEDB immunogenicity prediction tool [24]).

### 2.7. Analysis of Conservation

Selected epitopes’ conservation was evaluated as follows: (1) complete NP sequences from human H1N1, H3N2, and IBV strains were aligned using AliView (version 1.28), with duplicate sequences and poorly aligned regions removed; (2) the Site Counter program in Bioaider (version 1.314) [25] calculated amino acid residue types and the most common residue frequency at each position within selected peptides; (3) WebLogo [26] visualized peptide amino acid compositions to reflect sequence preferences; and (4) peptides with all residues matching sequence logos were deemed conserved and selected for MEMV candidate construction.

### 2.8. Construction of Multi-Epitope mRNA Vaccine

MEMV candidates were constructed with the following modular design: (1) conserved HTL/CTL epitopes joined by flexible GPGPG/AAY linkers [27]; (2) B-cell epitopes linked via KK linkers for structural stability [28]; (3) PADRE (a universal T-cell epitope) added to the N-terminus via an EAAAK linker [29], and tPA signal peptide [30] connected to PADRE using a GPGPG linker to enhance immunogenicity and presentation; (4) A CMV 5′UTR and hGH 3′UTR [31] frame the coding sequence for enhancing mRNA stability; and (5) A C-terminal 6 × His-tag was appended for potential in vitro protein detection.

### 2.9. Population Coverage Analysis

Population coverage of the selected CTL and HTL epitopes (with their corresponding HLA alleles) was analyzed using the IEDB Population Coverage Tool [32]. With default parameters, coverage was analyzed across 16 geographical regions, considering combined HLA class I and class II alleles.

### 2.10. Prediction of Antigenicity, Allergenicity, Toxicity, and Solubility

MEMV’s antigenicity was assessed via VaxiJen 2.018 with a 0.5 threshold. Allergenicity was evaluated using AllerTOP v.2.0, which achieves 85.3% accuracy in five-fold cross-validation [19]. Toxicity was predicted by ToxinPred2 [33], employing a Random Forest algorithm with a 0.5 threshold. Solubility was assessed via Protein-Sol [34], where a value > 0.45 indicates better solubility than average soluble *E. coli* proteins.

### 2.11. Physicochemical Properties and Structural Prediction and Optimization

MEMV’s physicochemical properties (amino acid count, molecular weight, pI, aliphatic index, instability index, GRAVY, and half-life) were assessed via Expasy Protparam [35]. Transmembrane topology, helix, folds, and domain recognition were predicted using PSIPRED [36], and peptide secondary structure was predicted via Prabi servers [37]. 3D models were generated with AlphaFold3 [38], and the rank 1 model of each candidate was optimized using GalaxyRefine [39] to enhance accuracy.

### 2.12. Structure Validation

The ProSA-web server [40] was used to evaluate the quality of MEMV 3D structures by calculating a Z-score, which reflects the deviation of the structures from naive proteins of similar size. Z-scores within the range of naturally occurring proteins (typically negative) indicate stable conformations, while Z-scores > 0 indicate potential structural errors or unstable regions. UCLA-DOE LAB SAVES v6.0 [41], specifically the ERRAT85 and PROCHECK86 servers, was employed to predict high-resolution crystal structures via non-bonded atom-atom interactions and generate Ramachandran diagrams.

### 2.13. Identification of Conformational B-Cell Epitopes

Most B-cell epitopes are conformational, composed of 1–5 discontinuous amino acid segments that are spatially proximal, forming antibody interaction sites [42] and inducing adaptive immunity [43]. Discontinuous epitopes were predicted using the ElliPro server [44] and visualized in NP’s 3D structure via PyMOL(Version 3.0.0).

### 2.14. Molecular Docking

TLR3 (PDB ID: 2A0Z) was obtained from MMDB, stripped of ligands in PyMOL, and docked to MEMVs via AlphaFold338 to generate stable complexes. Key contacts (H-bonds and salt bridges) were identified with PISA [45] and visualized in PyMOL.

### 2.15. Molecular Dynamics Simulation

GROMACS 2022 was employed to run 100 ns molecular dynamics simulations of the three MEMV–TLR3 complexes [46]. After energy minimization (50 k steps), each system—built with the AMBER99SB-ILDN force field, solvated in a 10 nm^3^ TIP3P water box (1.2 nm padding) and neutralized with NaCl—was equilibrated for 100 ps NVT (300 K) and 100 ps NPT (1 bar) using a Langevin thermostat and Berendsen barostat. Production under NPT (300 K, 1 bar) applied PME electrostatics, 1.0 nm cut-offs, P-LINCS constraints, and a 2 fs time step (frames 10 ps). RMSD, RMSF, Rg, buried area, and H-bond occupancy were extracted with GROMACS tools; binding free energies and per-residue contributions were calculated with gmx_MMPBSA (MM/PBSA), and solvent-accessible surface areas with gmx sasa.

### 2.16. Immune Simulations

The C-ImmSim server, an immune system analysis platform, was used to evaluate MEMV candidates’ ability to induce immune cells to produce specific antibodies and cytokines, simulating B and T lymphocyte responses to virtual vaccination [47]. Parameters were set as: Random Seed = 12345 (ensuring reproducibility); Simulation Volume = 10 (matching lymphoid tissue physiology); Simulation Steps = 1000; and HLA selection: A0101, A0201, B0702, B0801, DRB10101, DRB1501 (top global prevalent alleles per the Allele Frequency Net Database) [47]. Two injections of 1000 antigens were simulated at 4-week intervals. With each time step corresponding to 8 h, simulation periods were set at 1 and 84.

## 3. Results

### 3.1. Selection of Immunodominant B-Cell Linear Epitopes

**Figure 1:**
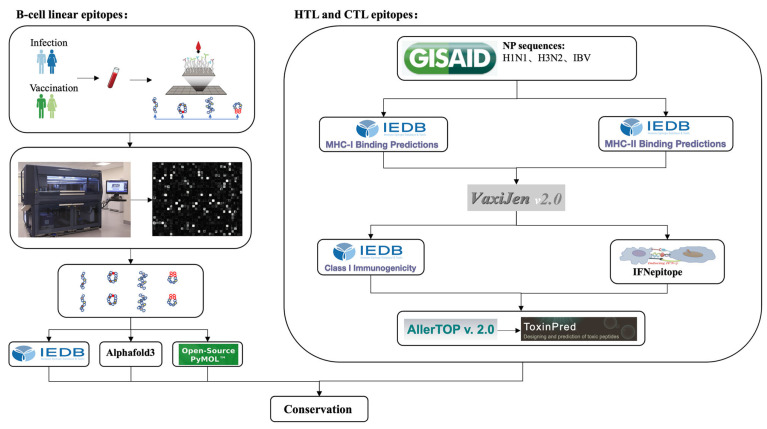
Schematic of the clinical serum-anchored epitope selection and MEMV design pipeline. Sera from influenza-infected (infection) and quadrivalent influenza vaccine-vaccinated (vaccination) cohorts were used to screen differential NP peptides via antibody-peptide microarray; subsequent bioinformatic analysis identified immunodominant B-cell linear, HTL, and CTL epitopes for the construction of MEMV candidates. Adapted from “In silico design of a broad-spectrum multiepitope vaccine against influenza virus”, by Lifang Yuan et al., 2024 [12], International Journal of Biological Macromolecules, 254, p. 12,8071. Copyright 2024 by Elsevier. Reprinted with permission.

**Figure 2:**
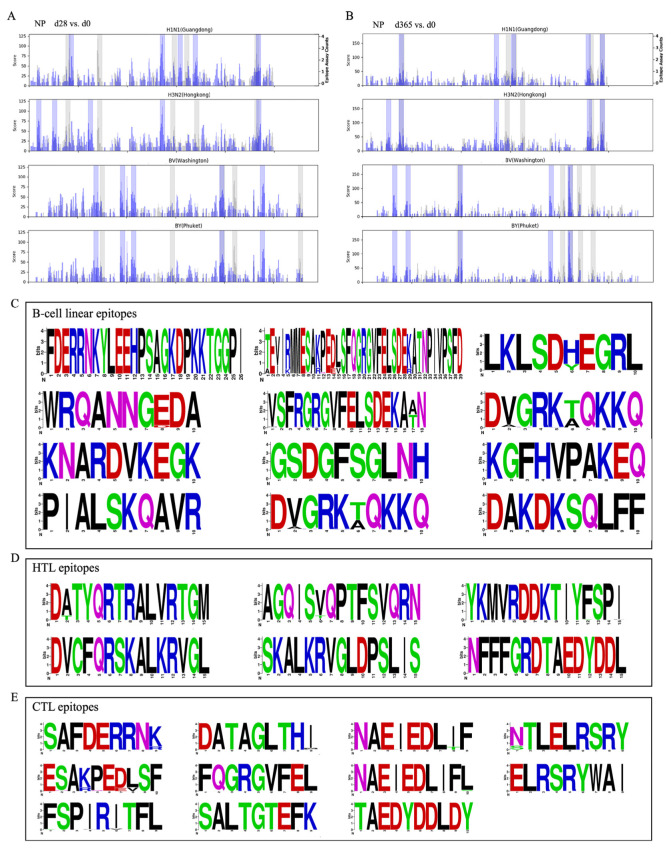
Integrated analysis of differentially expressed NP peptides and conserved epitope sequence logos from influenza A/B viruses. (**A**,**B**) Longitudinal sera (d0 vs. d28/d365) were used to identify epitopes with short-term and long-term immune responses. NP peptides from H1N1, H3N2, and IBV were ranked by *p*-value (*t*-test) at d28 and d365 versus d0 in QV (quadrivalent influenza vaccine recipients: blue) and IP (influenza patients: gray) sera, with the top five hits highlighted. (**C**–**E**) Sequence logos of conserved B-cell linear, HTL, and CTL epitopes: y-axis = amino acid residue conservation frequency, x-axis = peptide position.

The selection of immunodominant B-cell linear epitopes was anchored in clinical serum data from antibody-peptide microarray screening, ensuring the resulting epitopes are functionally relevant to human immune responses. Figure 1 outlines the selection pipeline. Antibody-peptide microarrays screened 36 sera (QIV-vaccinated and infected cohorts) at d0, d28, and d365; fluorescence intensity data (Table S1) identified the top five differential NP peptides per subtype (H1N1, H3N2, IBV) via *t*-test (Figure 2A,B, Table S2). After cross-checking with IEDB-annotated IAV-NP B-cell epitopes (Table S3) and full-length NP alignments, two well-aligned peptides (FDERRNKYLEEHPSAGKDPKKTGGPI and TEIIRMMESARPEDVSFQGRGVFELSDEKATNPIVPSFD) were merged into a single conserved epitope (VSFRGRGVFELSDEKAAN), and the unmapped top five peptides were retained. Further bioinformatic filtering retained antigenic (>0.5), non-allergenic, non-toxic, and surface-exposed epitopes, yielding 12 final B-cell linear epitopes (Table S4).

### 3.2. Selection of Immunodominant HTL and CTL Epitopes

HTL and CTL epitopes from H1N1, H3N2, and IBV NP with a percentile rank <0.5 were initially selected. Antigenicity analysis via VaxiJen 2.0 retained HTL epitopes with scores >0.5 and CTL epitopes with scores >1.0. Additional filtering (CTL immunogenicity >0, HTL IFN-γ positive, non-allergenic, and non-toxic) identified 6 HTL and 11 CTL epitopes for further analysis (Table S5).

### 3.3. Conservation Analysis

NP sequences from prevalent human H1N1 (1877), H3N2 (2301), and IBV (1762) strains were retrieved from the Influenza Virus Database, with duplicates removed. The Site Counter program in Bioaider (version 1.314) [25] calculated amino acid residue types and frequencies at each position within selected peptides. Amino acid frequencies (Table S6), proportions (Table S7), maximum proportions, and mutation rates (Table S8) were determined; full-length mutations are shown in Figure S1. Conservation analysis of 12 B-cell, 6 HTL, and 11 CTL epitopes identified significant mutations in 3 B-cell (TEIIRMMESARPEDVSFQGRGVFELSDEKATNPIVPSFD, WRQANNGKDA, DVGRKAQKKQ), 2 HTL (AGQISIQPTFSVQRN, DVCFQRSKALKRVGL), and 5 CTL (DATAGLTHM, NAEFEDLTF, STLELRSRY, ESARPEDVSF, FQGRGVFEL) epitopes. The remaining nine B-cell, four HTL, and six CTL epitopes (Table S9) were highly conserved across H1N1, H3N2, and IBV strains (Figure 2C–E) and selected for MEMV construction.

### 3.4. Construction Strategy

**Figure 3:**
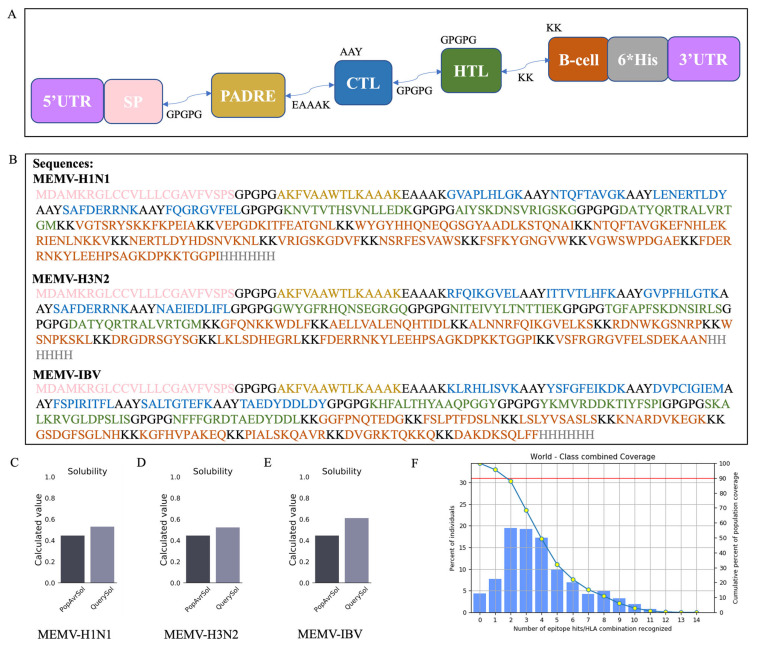
Design and global HLA population coverage prediction of MEMV candidates from the clinical serum-anchored pipeline. (**A**) Schematic composition of MEMV candidates, including tPA signal peptide, PADRE, HTL/CTL epitopes, and B-cell linear epitopes. (**B**) Amino acid sequences of MEMV-H1N1, -H3N2, and -IBV, color-coded by functional module. (**C**–**E**) Protein-Sol predicted solubility profiles of the three MEMV candidates. (**F**) Global HLA class I/II combined population coverage of the epitopes in the MEMV design pipeline.

The construction strategy for three MEMV candidates (MEMV-H1N1, MEMV-H3N2, and MEMV-IBV) is outlined in Figure 3A,B. B-cell linear epitopes were connected using KK linkers, HTL and CTL epitopes with GPGPG and AAY linkers, respectively. PADRE was added to the N-terminus via an EAAAK linker, and the signal peptide was connected to PADRE using a GPGPG linker. 5′UTR and 3′UTR elements were incorporated to enhance mRNA stability, and a C-terminal 6 × His-tag was appended for potential in vitro detection.

### 3.5. Results of the Antigenicity, Allergenicity, Toxicity, and Solubility Prediction

VaxiJen 2.0-predicted antigenicity scores were 0.6497 (MEMV-H1N1), 0.6025 (MEMV-H3N2), and 0.7311 (MEMV-IBV)—all above the 0.5 threshold. AllerTOP v.2.0 confirmed non-allergenicity, and ToxinPred2 predicted non-toxicity for all candidates. Scaled solubility values were 0.530 (MEMV-H1N1), 0.523 (MEMV-H3N2), and 0.611 (MEMV-IBV) (Table 1), all exceeding the 0.45 threshold for high solubility (Figure 3C–E). These results indicated the MEMV sequences derived from the pipeline have favorable biophysical properties for potential in vitro validation.

### 3.6. Results of Population Coverage Analysis

To account for HLA allele variability across ethnicities and regions, population coverage of selected HTL/CTL epitopes with their HLA alleles was evaluated. Combined HLA class I/II alleles showed 95.63% global coverage (Figure 3F), spanning 16 regions and 109 countries (Table S10)—confirming the pipeline’s applicability to diverse ethnic populations.

### 3.7. Prediction of Physicochemical Properties

Key physicochemical properties are summarized in Table 1: MEMV-H1N1 (351 aa, 38.76 kDa, pI 9.89), MEMV-H3N2 (331 aa, 36.56 kDa, pI 10.05), and MEMV-IBV (312 aa, 33.94 kDa, pI 9.68). All candidates exhibited favorable stability (instability index < 40) and hydrophobicity (negative GRAVY scores), with in vitro half-lives of 30 h (human reticulocytes), >20 h (yeast), and >10 h (*E. coli*)—supporting potential in vitro expression and in vivo persistence.

### 3.8. Structure Analysis

**Figure 4:**
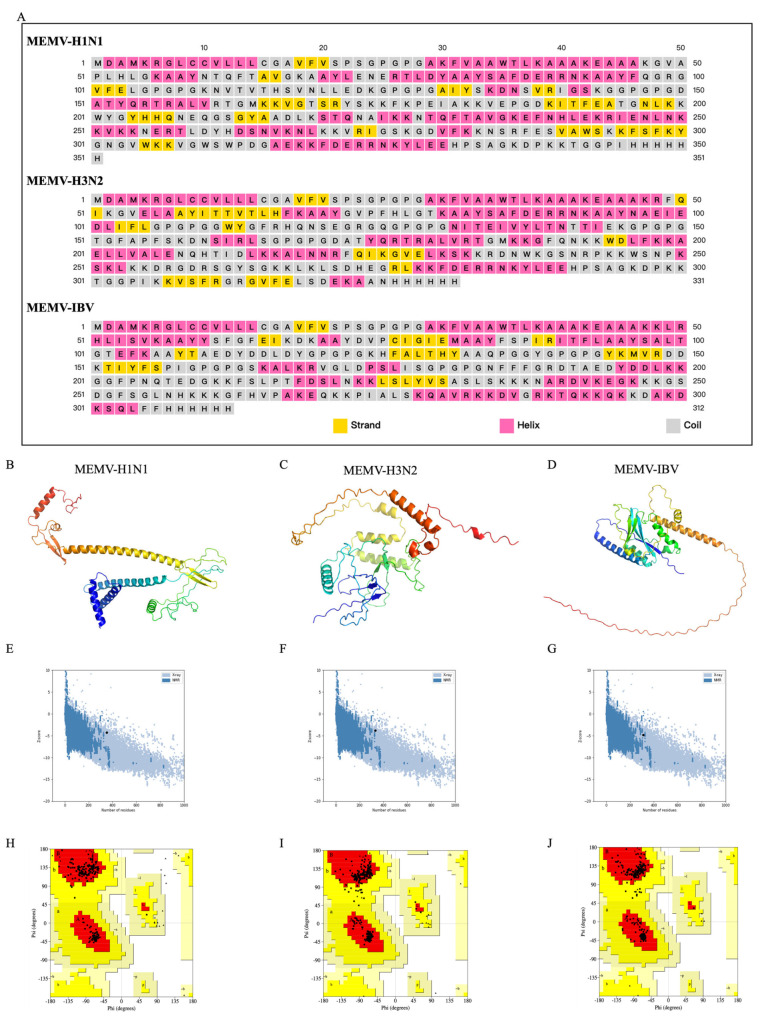
AlphaFold3-based 3D modeling and structural validation of pipeline-derived MEMV candidates. (**A**) Secondary structure of MEMV candidates: β-strands (yellow); α-helices (pink); random coils (gray). (**B**–**D**) Refined AlphaFold3 3D models of MEMV candidates. (**E**–**G**) ProSA Z-scores of the optimized 3D models: −4.91, −3.77, and −4.80. (**H**–**J**) Ramachandran plots for structural validation: favored (red); allowed (deep-yellow); generously allowed (light-yellow); outliers (white); glycines (triangles).

PSIPRED/Prabi analysis revealed secondary structures (Figure 4A): MEMV-H1N1 (35.61% α-helices, 21.94% extended strands, 42.45% random coils); MEMV-H3N2 (41.99% α-helices, 13.60% strands, 44.41% coils); and MEMV-IBV (41.35% α-helices, 10.09% strands, 47.76% coils). Abundant random coils increased epitope accessibility, which is favorable for immune recognition. 3D structures (AlphaFold3; Figure 4B–D) were refined via GalaxyRefine. Optimal models (MEMV-H1N1: model 1; MEMV-H3N2/IBV: model 5) were selected based on GDT-HA, RMSD, and MolProbity scores. ProSA-web Z-values were −4.91 (MEMV-H1N1), −3.77 (MEMV-H3N2), and −4.80 (MEMV-IBV) (Figure 4E–G). ERRAT quality factors were 89.84, 95.15, and 92.082, respectively. PROCHECK Ramachandran analysis (Figure 4H–J) showed most residues in favored regions: 94.4% (MEMV-H1N1), 96.5% (MEMV-H3N2), and 94.7% (MEMV-IBV), with minimal disallowed residues—confirming stable conformations.

### 3.9. Conformational B-Cell Epitopes

ElliPro server analysis identified conformational B-cell epitopes in all MEMV candidates: five epitopes (50 residues) in MEMV-H1N1, six epitopes (36 residues) in MEMV-H3N2, and eight epitopes (58 residues) in MEMV-IBV (Table S11 and Figure S2). These conformational epitopes complement B-cell linear epitopes, expanding the pipeline’s coverage of immunogenic regions.

### 3.10. Molecular Docking Between the MEMV Candidates and TLR3

**Figure 5:**
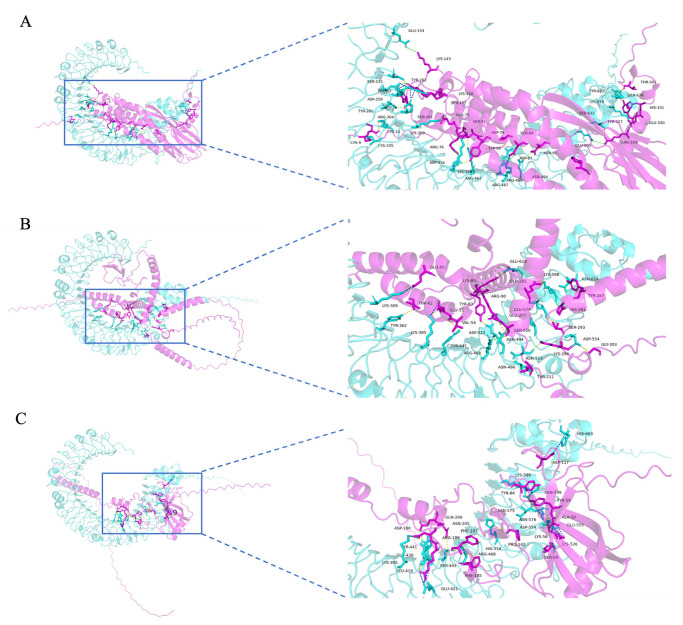
Molecular docking between pipeline-derived MEMV candidates and TLR3. (**A**–**C**) 3D docking complexes of MEMV-H1N1, MEMV-H3N2, MEMV-IBV (magenta) with TLR3 (cyan), respectively. Magnified binding interfaces show key interacting residues: hydrogen bonds (yellow), salt bridges (blue), and mixed hydrogen bond–salt bridge interactions (green).

Molecular docking was performed to assess the potential immune activation of MEMV candidates and TLR3—a pattern recognition receptor critical for antiviral immunity via dsRNA recognition and type I IFN/inflammatory cytokine production [48,49]. Docking results revealed stable interactions: MEMV-H1N1-TLR3 (Figure 5A) showed 25 hydrogen bonds (involving residues Cys9-Cys335, Arg76-Asp416, Tyr80-Asp416, Asn91-Asn494, Ser137-Tyr262, Arg139-Asp259, Arg139-Ser235, Arg139-Ser261, Lys143-Glu154, Arg323-Ser632, Arg323-Glu605, Tyr327-Ser674, His331-Ser674, Cys10-Arg304, Ser21-Arg463, Phe63-Tyr281, Glu75-Lys309, Asp79-Lys395, Ser84-Arg467, Asp87-Arg468, Lys134-Tyr262, Thr341-Tyr662, and Thr341-Ser674) and 19 salt bridges (including Arg76-Asp416, Arg139-Glu285, Arg139-Asp259, Lys143-Glu154, Arg323-Glu605, Glu75-Lys309, Asp79-Lys395, Asp87-Arg468, and Glu330-Lys676); MEMV-H3N2-TLR3 (Figure 5B) showed 16 hydrogen bonds (involving residues Thr62-Tyr362, Lys80-Glu618, Tyr83-Asp515, Arg90-Asn494, His291-Lys598, Ser293-Leu574, Lys296-Asn519, Lys296-Ser550, Gly303-Asp554, Glu43-Lys309, Val54-Tyr441, Glu55-Lys395, Glu201-Lys598, Glu207-Arg468, Thr211-Asn496, and Tyr287-Asn624) and 8 salt bridges (including Lys80-Glu618, Glu43-Lys309, Glu55-Lys395, Glu201-Lys598, and Glu207-Arg468); and MEMV-IBV-TLR3 (Figure 5C) showed 16 hydrogen bonds (involving residues Lys56-Glu555, Lys56-Glu554, Tyr59-Lys598, Tyr59-Asn575, Tyr84-Asn599, Arg189-Leu419, Asn205-Ser443, Gln206-Tyr441, Gln206-Glu439, Ser54-Lys526, Lys56-Asn576, Lys58-Asn576, Pro142-His518, Phe185-Arg468, Phe187-Arg468, and Asp190-Lys395) and 10 salt bridges (including Lys56-Glu555, Lys56-Glu554, Arg189-Glu421, Asp117-His663, and Asp190-Lys396). Consistent with a 100 ns molecular dynamics simulation, the multiple hydrogen bonds and salt bridges confirmed favorable conformational compatibility between MEMV candidates and TLR3. These results provide computational evidence for MEMV’s potential to synergize with TLR3-mediated innate immunity, laying a structural foundation for efficient antigen presentation and adaptive immune responses.

### 3.11. Results of Molecular Dynamics Simulation

**Figure 6:**
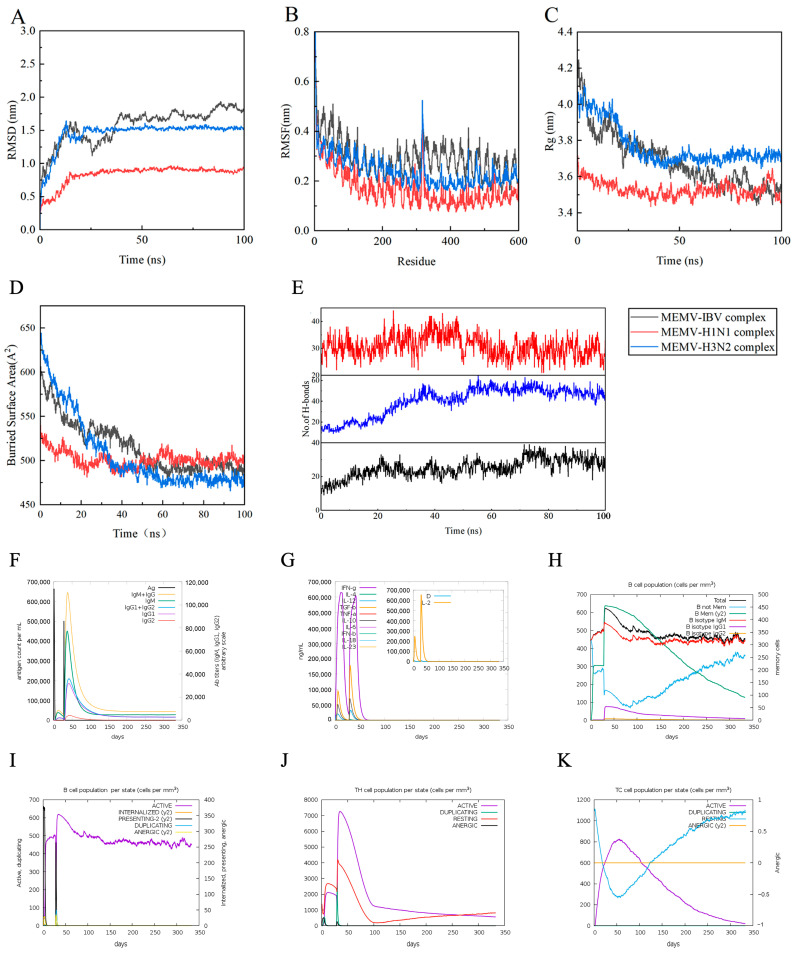
Molecular-dynamics stability and in silico immune response profiles of MEMV-TLR3 complexes from the design pipeline. (**A**) Root Mean Square Deviation (RMSD) of MEMV-H1N1-TLR3, MEMV-H3N2-TLR3, and MEMV-IBV-TLR3 complexes over 100 ns. (**B**) Root Mean Square Fluctuation (RMSF) plot of the three complexes. (**C**) Radius of gyration (Rg) variation in the complexes. (**D**) Buried surface area of the MEMV-TLR3 binding interfaces. (**E**) Dynamic changes in the number of intermolecular hydrogen bonds (H-bonds) in the complexes. (**F**–**K**) C-ImmSim immune simulation results after two MEMV injections at a 4-week interval: (**F**) total Ig and Ig subclass titers; (**G**) key cytokine secretion levels (inset: IL-2 and macrophage signal D); (**H**) total B-cell kinetics; (**I**) active B-cell secretion (purple); (**J**) CD4^+^ T-helper (Th) cell subsets; and (**K**) CD8^+^ cytotoxic T (Tc) cell activation states.

The 100 ns molecular dynamics simulations of the three MEMV–TLR3 complexes revealed rapid equilibration and sustained stability (Figure 6A–E). RMSD plateaued within 20 ns for MEMV-H1N1-TLR3 and MEMV-H3N2-TLR3, and by 40 ns for MEMV-IBV-TLR3 (Figure 6A). RMSF profiles showed only minor fluctuations at defined residues (e.g., 150, 252, 330 in H1N1; termini in H3N2; and 259 in IBV) without compromising overall integrity (Figure 6B). Radius of gyration remained compact (3.69, 3.44, and 3.55 nm, respectively) throughout the trajectory (Figure 6C). Buried surface area increased or remained stable after ~50 ns, reflecting tighter hydrophobic packing (Figure 6D), while intermolecular hydrogen bonds averaged 40.5 ± 13.6, 30.8 ± 4.1, and 25.1 ± 5.4 for MEMV-H1N1-TLR3, MEMV-H3N2-TLR3, and MEMV-IBV-TLR3 complexes, respectively (mean ± SD; Figure 6E). These simulation results confirmed that the MEMV–TLR3 complexes have long-term stability, supporting the pipeline’s potential to induce consistent immune activation.

### 3.12. Immune Responses for Vaccine Efficacy

**Figure 7:**
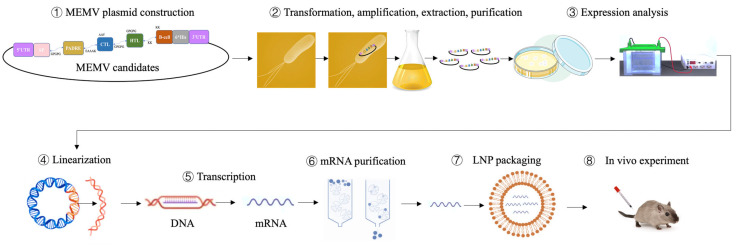
Schematic workflow for the production of MEMV candidates via the clinical serum-anchored computational design pipeline.

In silico immunization with two MEMV injections (via C-ImmSim) boosted IgM + IgG, IgM, IgG1 + IgG2, IgG1, and IgG2 titers while reducing antigen load (Figure 6F). IFN-γ, TGF-β, and IL-10 peaked, with smaller rises in IL-12 and IL-4 (Figure 6G). B-lymphocyte counts increased, yielding IgM-secreting and memory B cells alongside activated B-cell populations (Figure 6H–I). TH cells (active, resting, proliferating) expanded after each injection, whereas active and proliferating TC cells rose then fell (Figure 6J–K). Additional immune subsets are presented in Figure S3. Overall, in silico simulation showed MEMVs could elicit robust, durable immune response characteristics, providing positive predictive evidence for subsequent experimental validation of the pipeline. A production workflow for the pipeline is outlined in Figure 7. Notably, C-ImmSim simulation results are dependent on parameter settings and cannot fully replicate in vivo immune complexity. Thus, the predicted immune characteristics require experimental validation.

## 4. Discussion

Annual influenza vaccination remains the primary, cost-effective prophylaxis endorsed by the World Health Organization [50,51], yet its effectiveness is eroded by continual antigenic drift that generates vaccine–strain mismatch [5]. A broad-spectrum vaccine design strategy capable of targeting diverse influenza A/B strains is therefore urgently needed. A key strength of this study is the experimental anchoring of vaccine design in longitudinal clinical serological data—a distinguishing feature that sets it apart from purely in silico studies and even most existing experimental and computational vaccine designs. Using NP as a conserved immunogen (less prone to antigenic drift than HA/NA), we first mapped immunodominant B-cell linear epitopes via high-density antibody-peptide microarrays, leveraging 36 longitudinal sera from 12 well-characterized human cohorts (6 quadrivalent inactivated influenza vaccine recipients and 6 laboratory-confirmed influenza patients). As reported, original antigenic sin occurs when pre-existing influenza immunity distorts subsequent immune responses, leading to heterogeneous reactions to specific strains among individuals with different immune backgrounds [52,53].

To avoid this interference, we strictly excluded participants with a history of influenza infection or vaccination in the past three years, ensuring that the immune responses captured in our longitudinal serum analysis reflect de novo reactions to vaccination or acute infection. Moreover, two critical timepoint comparisons ensured epitope quality: (1) d28 vs. d0 to capture “acute immune response epitopes” (potent peptides reflecting early post-vaccination/infection reactivity) and (2) d365 vs. d0 to identify “long-lived immune response epitopes” (durable peptides indicative of sustained immunity). This dual-timepoint experimental workflow ensured the selected epitopes are not just computationally predicted but functionally reactive in human hosts—with both short-term immunogenicity (d28) and long-term persistence (d365), these two prerequisites for effective vaccine development are rarely addressed simultaneously in comparable studies relying on single-timepoint samples.

Notably, several B-cell epitopes identified in our experimental screening are absent from IEDB (Table S4), indicating novelty and highlighting the value of direct serological mapping in discovery. Consistent with the previous literature reports, naturally induced antibodies are sustained [54], whereas vaccine-elicited responses are shorter-lived and narrower [55]. This underscores the advantage of integrating both infection-derived and vaccine-derived epitopes into the MEMV design pipeline—balancing robust short-term antibody reactivity (at d28) and long-term antibody persistence (at d365).

These experimentally validated B-cell epitopes (selected for both d28 potency and d365 durability) were then combined with in silico-predicted conserved HTL and CTL epitopes (from NP/HA/NA) targeting HLA-A/B alleles [56,57]. Conservation analysis yielded nine B-cell and ten T-cell NP epitopes that are antigenic, non-allergenic, non-toxic, and highly conserved; these were integrated with previously validated HA- and NA-derived epitopes [13] to construct the MEMV design pipeline.

Recently, mRNA vaccines have displayed distinct advantages over classical platforms—speed, adaptability, and safety [8]—and have already incorporated conserved HA/NA to elicit promising protective effects in animal models [58,59]. Extending this concept, we rationally designed three mRNA constructs (MEMV-H1N1, -H3N2, and -IBV) that co-encode experimentally validated dual-timepoint B-cell linear epitopes (screened via d0/d28/d365 longitudinal sera) and T-cell epitopes derived from HA, NA, and NP, covering the full spectrum of conserved influenza antigens. This integrated epitope design addresses a critical challenge in broad-spectrum vaccine development: the inherent trade-off between cross-subtype protection and subtype-specific neutralizing potency, as highlighted by a representative HA-based broad-spectrum vaccine study [60].

That study used scrambled HA mutants to redirect antibody responses to conserved epitopes, achieving broad protection but weaker subtype-specific neutralization—an issue stemming from the immune system allocating resources to diverse antigenic determinants. In contrast, our strategy leverages the synergy between humoral and cellular immunity by harnessing the complementary roles of epitopes from three antigens (HA, NA, and NP): B-cell epitopes from all three antigens induce subtype-specific neutralizing antibodies, while conserved HTL/CTL epitopes from the same antigens elicit CD4^+^ helper T-cell and CD8^+^ cytotoxic T-cell responses. Importantly, T-cell responses targeting conserved epitopes—especially from NP, a conserved internal protein with >90% sequence conservation across influenza A and B strains—provide robust protection against severe disease across divergent subtypes without impairing the immune system’s capacity to target individual strains.

To enhance immunogenicity, PADRE was added at the N-terminus via an EAAAK linker [29], and a signal peptide was appended through a GPGPG linker [30]. Flexible GPGPG linkers were used between HTL epitopes, and AAY linkers between CTL epitopes, to optimize proteasomal cleavage and MHC presentation [61]. Moreover, our epitope selection—anchored in longitudinal human serum data (d0/d28/d365)—ensures the selected epitopes strike a balance between broad cross-reactivity and sustained subtype-specific immune memory, mitigating the trade-off observed in purely structure-based modification strategies. Building on these insights, we will design parallel broad-spectrum multi-epitope mRNA constructs and subtype-specific mRNA constructs to compare their in vitro neutralizing activity against influenza strains and in vivo protective efficacy in animal models. This will allow us to optimize the epitope combination ratio and achieve a better balance between broad coverage and subtype-specific potency.

As expected, the physicochemical characteristics of these multi-epitope mRNA vaccines (MEMVs) indicated that they are suitable vaccine candidates with excellent thermostability. Physicochemical profiling confirmed thermostability; secondary-structure prediction revealed abundant coil regions that increase flexibility and antibody access. Docking and 100 ns MD simulations demonstrated stable, high-affinity engagement with TLR3, as evidenced by convergent RMSD, Rg, and persistent interfacial contacts. C-ImmSim modeling predicted robust immunity after two injections: marked elevations in IgM + IgG, IgM, IgG1 + IgG2, IgG1 and IgG2, and parallel rises in IFN-γ, TGF-β, IL-10, IL-12, and IL-4. B-lymphocyte counts expanded with each boost, accompanied by elevated IgM-secreting cells, memory B cells, and active B-cell populations—indicative of potential durable immunological memory.

These results support the use of the clinical serum-anchored computational pipeline as a rapid, cost-effective route to broad-spectrum influenza vaccine design. Compared with traditional purely in silico platforms, this pipeline offers three key advantages: (1) Clinical relevance: Epitopes are anchored in human serum data, reducing discrepancies between prediction and in vivo efficacy; (2) all steps rely on public databases (IEDB, GISAID) and tools (AlphaFold3, GROMACS), enabling reuse by other researchers; (3) Efficiency: Shortens the preliminary design cycle by merging clinical screening and computational optimization, avoiding trial-and-error in epitope selection.

Nevertheless, three methodological limitations remain: (1) in vivo mRNA stability and delivery efficiency need to be further optimized; (2) epitope competition may occur in vivo to blunt sub-dominant immune responses; in addition, the small sample size of 12 human cohorts limits the direct generalization of epitope prevalence to the global population; (3) NP was used as the core antigen in this study, with only B-cell linear epitopes experimentally verified, while HTL and CTL epitopes were predicted in silico without further experimental validation. NP-specific B-cell epitopes lack neutralizing activity, and their theoretical roles in promoting T-cell activation and immune memory also remain to be confirmed.

Future work will construct proteins to verify the immunogenicity of NP-derived HTL and CTL epitopes, validate the supportive roles of NP B-cell epitopes in T-cell activation and immune memory, test the neutralizing activity of HA/NA-derived epitopes, use animal models to evaluate comprehensive immune responses and cross-protection, optimize mRNA ratios and adjuvantation to achieve balanced and durable immunity, and validate the pipeline with larger and more diverse serum cohorts to confirm epitope generalizability and population representativeness.

This study is a methodological exploration of in silico influenza vaccine design. The pipeline’s value lies in providing a reusable framework for rapid, clinically relevant vaccine design, and the MEMV sequences are targets for subsequent experimental validation. In vivo experiments are critical for vaccine validation, so we have included in vivo validation as a key future direction, and the pipeline’s in silico results (stable TLR3 binding and robust immune simulation) provide a strong basis for these experiments.

## 5. Conclusions

This study established a clinical serum-anchored computational design pipeline for broad-spectrum influenza multi-epitope mRNA vaccines (MEMVs), with three core methodological contributions: developing a novel workflow integrating longitudinal human serum screening (d0/d28/d365) and multi-dimensional computational optimization, which addresses the clinical data gap in pure in silico design and ensures selected epitopes have both short-term immunogenicity and long-term persistence; providing standardized templates for MEMV construction and epitope selection; and verifying the pipeline’s predictive validity through comprehensive in silico assays. Future research will focus on the experimental validation of epitope immunogenicity, in vivo antibody profiles, and cross-protective efficacy, advancing the application of this methodological strategy in broad-spectrum vaccine development.

## Figures and Tables

**Table 1:** The basic features, physicochemical properties, and half-life (hours) of the MEMV candidates.

	Candidates		
**Parameters**	MEMV-H1N1	MEMV-H3N2	MEMV-IBV
**Basic features**			
Antigenicity	0.6497	0.6025	0.7311
Allergenicity	Non-allergen	Non-allergen	Non-allergen
Toxicity	Non-toxin	Non-toxin	Non-toxin
Solubility	0.530	0.523	0.611
**Physicochemical properties**			
Number of amino acids	351	331	312
Molecular weight (kDa)	38.76	36.56	33.94
Theoretical isoelectric point (pI)	9.89	10.05	9.68
Aliphatic index	58.97	64.05	64.23
Instability index	16.39	22.11	29.17
GRAVY score	−0.792	−0.747	−0.523
**Half-life (hours)**			
Mammalian reticulocytes, in vitro	30	30	30
Yeast, in vivo	>20	>20	>20
*Escherichia coli*, in vivo	>10	>10	>10

## Data Availability

The datasets utilized in this study can be obtained from the following sources: the GISAID Influenza Virus Database (gisaid.org), the NCBI Influenza Virus Resource (https://www.ncbi.nlm.nih.gov/genomes/FLU/Database/nph-select.cgi?go=database, accessed on 25 February 2025), and IEDB (https://www.iedb.org/, accessed on 18 December 2024).

## Supplementary Materials

The following supporting information can be downloaded at: https://www.mdpi.com/article/10.3390/biology15040357/s1. Figure S1: Conservation analysis of the selected full-length NP sequences of circulating influenza viruses. Figure S2: The conformational epitopes predicted by the ElliPro server. Figure S3: Immune simulation results of MEMVs. Table S1: The relative fluorescence score for each peptide on days 28 and 365 after vaccination, comparing them to the baseline defined as day 0 before vaccination. Table S2: The top 5 peptides of NP with significant difference fluorescence scores in each influenza virus subtype. Table S3: The positive B-cell linear epitopes of NP of selected influenza virus subtypes were downloaded from the IEDB database. Table S4: The B-cell linear epitopes selected for further conservation analysis. Table S5: The HTL and CTL epitopes selected for further conservation analysis. Table S6: The count of amino acids present at specific sites in NP sequences of H1N1, H3N2 and IBV was calculated using the Site Counter program in Bioaider from all sequences. Table S7: The site proportions of amino acids in NP sequences of all H1N1, H3N2 and IBV were calculated using the Site Counter program in Bioaider. Table S8: The maximum proportions and mutations of amino acids at the sites in NP sequences of all H1N1, H3N2, and IBV were calculated using the Site Counter program in Bioaider. Table S9: The B-cell linear epitope, HTL, and CTL epitopes used for MEMV candiates caonstruction. Table S10: Population Coverage analysis of the final vaccine construct using the population coverage analysis tool of the IEDB database by keeping the default parameters on (109 countries covering 16 different geographical regions). Table S11: The conformational B cell epitope residues of the MEMV candidates predicted by ElliPro server.
