# Supplementary material for: Clinical Serum-Anchored Computational Design Pipeline for a Broad-Spectrum Influenza Multi-Epitope mRNA Vaccine
Source: Biology (Basel). 2026 Feb 19;15(4):357. doi: 10.3390/biology15040357 (PMC12937623; doi:10.3390/biology15040357)
Supplement: Supplementary file 1 [file biology-15-00357-s001.zip › Supplemental Figures and Figure legend.pdf]

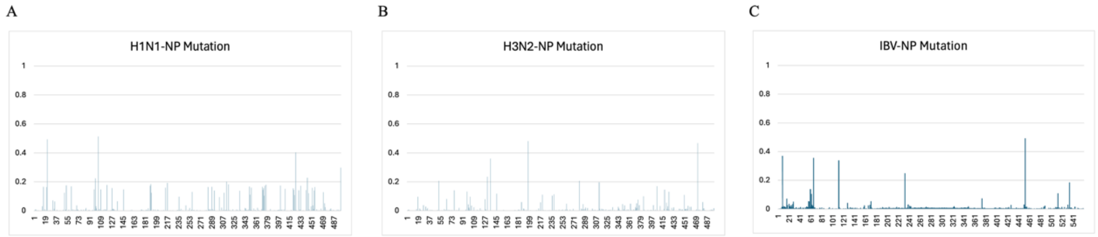


**Figure S1. Conservation analysis of the selected full-length NP sequences of circulating influenza viruses.**

Conservation analysis of full length NP sequences from circulating influenza viruses was performed using the Site Counter program in BioAider. The x-axis denotes NP peptide amino acid positions, and the y-axis represents amino acid conservation levels across all analyzed sequences.

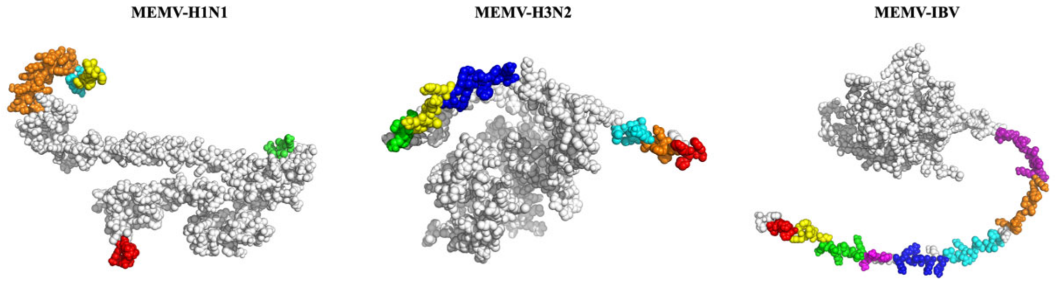


**Figure S2. The conformational epitopes predicted by the ElliPro server.**

Conformational B-cell epitopes of the three MEMV candidates (MEMV-H1N1, MEMV-H3N2, MEMV-IBV) were predicted using the ElliPro server.

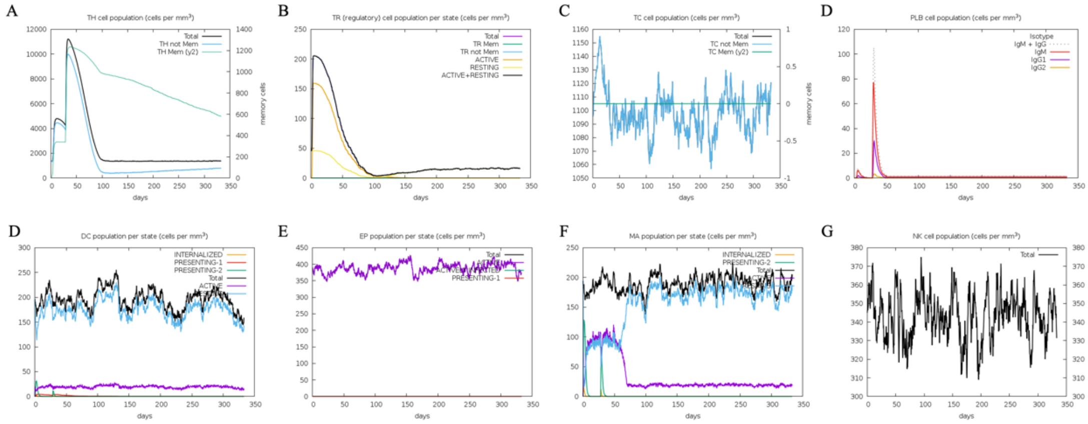


**Figure S3. Immune simulation results of MEMVs.**

In silico immune cell population dynamics of the three MEMV candidates were predicted with the C-ImmSim server. The plot shows dynamic changes in the populations of T helper (TH), T regulatory (TR), T cytotoxic (TC), plasmablasts (PLB), dendritic cells (DC), epithelial cells (EP), macrophages (MA), and natural killer (NK) cells after virtual MEMV immunization, with distinct subclasses colored differently.
